# Naphthalene-Based Aromatic Copolyesters Containing Polar Units with Improved Wettability and High Transparency for Biomedical Applications

**DOI:** 10.3390/polym18182212

**Published:** 2026-09-11

**Authors:** Alessandra Perrucci, Daniela Pollutri, Giulia Guidotti, Michelina Soccio, Piera Versura, Luigi Fontana, Nadia Lotti

**Affiliations:** 1 Civil, Chemical, Environmental and Materials Engineering Department, University of Bologna, 40131 Bologna, Italy; alessandra.perrucci2@unibo.it (A.P.); m.soccio@unibo.it (M.S.); nadia.lotti@unibo.it (N.L.); 2 IRCCS Azienda Ospedaliero-Universitaria di Bologna, 40138 Bologna, Italy; piera.versura@unibo.it (P.V.); luigi.fontana6@unibo.it (L.F.); 3 Ophthalmology Unit, Department of Medical and Surgical Sciences, (DIMEC), Alma Mater Studiorum University of Bologna, 40138 Bologna, Italy

**Keywords:** aromatic polyesters, poly(pentamethylene naphthalate), sulfonated copolyesters, hydrophilicity, transparency, biomedical applications

## Abstract

The development of novel high-performance materials is a key driver of technological progress in biomedicine. To date, polymeric materials have represented one of the most significant advances in addressing a broad range of challenges. Owing to their versatility and tunable properties, polymers have found countless applications, including in highly demanding biomedical fields, particularly when the availability of donor tissue is limited and transplantation is not always a viable option. To overcome these limitations, increasing research efforts have been devoted to the development of tailor-made biocompatible materials capable of restoring physiological functions. Within this context, the present research focuses on the synthesis and characterization of innovative aromatic polyesters containing polar comonomeric units to enhance their hydrophilicity. The reference homopolymer, poly(pentamethylene naphthalate), was chemically modified by incorporating different molar amounts of dimethyl 5-sulfoisophthalate subunits, with the aim of improving surface wettability and biological integration. In addition to high thermal stability and processability, the resulting materials exhibited good optical transparency, with low color saturation and a faint bluish hue, making them potentially suitable for biomedical applications, including the treatment of ocular tissues. Finally, biocompatibility was preliminarily assessed through in vitro cytotoxicity tests. Overall, these findings may lay the foundation for developing a new generation of materials capable of providing advanced solutions for biomedical applications.

## 1. Introduction

In the biomedical field, aromatic polyesters have emerged as promising biomaterials for many applications, including the treatment of damaged tissues, owing to their excellent mechanical performance, chemical stability, and the possibility of tailoring their surface and bulk chemistry to enhance biocompatibility and tissue integration. Recent developments in functional aromatic copolyesters have focused on improving hydrophilicity and cell–material interactions, enabling their use in advanced tissue engineering applications [1,2,3].

Within this class, the aromatic polyesters most commonly investigated to date for tissue repair are those containing a benzene ring, such as poly(ethylene terephthalate), PET [4,5,6], which is the most widely used aromatic polyester in medicine; poly(butylene terephthalate) (PBT) [7,8]; and poly(butylene isophthalate) (PBI). Their applications, either alone or in combination with other polymeric materials, range from the regeneration of hard tissues, such as bone, teeth, and ligaments, to the development of vascular grafts [9,10], wound healing, and drug delivery systems. Targeted surface modification also broadens the application window of aromatic polyesters, enhancing their antimicrobial and antioxidant activity, release efficiency, and biocompatibility [8,11,12]. For example, Khan et al. immobilized zinc oxide nanoparticles at different concentrations on PET fabrics to impart exceptional antibacterial and UV protection properties to the neat polymeric matrix for potential use in medical textiles [13].

Unlike terephthalic and isophthalic acid-based polyesters, naphthalene-2,6-dicarboxylic acid (NDCA)-based polyesters [14] have been investigated only sparsely for biomedical applications. From the chemical point of view, poly(alkylene naphthalate)s possess a molecular architecture analogous to that of poly(alkylene terephthalate)s, differing only in the replacement of the benzene ring with a naphthalate moiety. As these aromatic polyesters show enhanced chain rigidity and chemical and thermal stability [15,16,17], they have been investigated as promising high-performance materials, for example, in high-performance packaging applications [18], exploiting the rigidity of the naphthalene ring to improve thermal, mechanical, and barrier properties. Moreover, the large-scale availability of naphthalene-2,6-dicarboxylic acid, with a global market valued at USD 268 million in 2025 and forecast to increase at a 1.7% CAGR during the 2026–2034 period, has stimulated growing interest in this family of polyesters [19,20]. All these characteristics, together with high hydrolytic resistance, suggest that NDCA-containing copolyesters may represent promising candidates for future biomedical applications, though their effectiveness is yet to be tested.

Another important monomer used to prepare hydrophilic aromatic copolyesters is dimethyl 5-sulfoisophthalate (DMSI, or its sodium salt), which enables the introduction of pendant sulfonate groups into aromatic polyester chains. The resulting sulfonated copolyesters exhibit enhanced hydrophilicity, surface wettability, and water uptake. Examples reported in the literature include copolymers based on poly(ethylene terephthalate) [21], poly(ethylene furanoate) [22], poly(hexamethylene terephthalate) [23], poly(butylene adipate) [24], and poly(butylene succinate) [25,26], the latter also being investigated in terms of biocompatibility [27,28]. Indeed, DMSI is useful for promoting favorable cell–material interactions while preserving the excellent mechanical properties of the starting polyesters. Consequently, these sulfonated materials can be considered attractive candidates for biomedical applications, including tissue engineering scaffolds, ophthalmic devices, and drug delivery systems. To the best of our knowledge, the incorporation of this unit inside a naphthalate-based copolyester has not yet been reported in the literature. Lastly, obtaining transparent materials is essential for many biomedical applications, such as ophthalmology, but also microfluidics and diagnostic equipment, because it enables, for example, optical monitoring, light-based imaging, and real-time fluid tracking. Examples of polymeric materials already explored in the literature [29,30,31,32] include poly(vinyl alcohol) (PVA), poly(methyl methacrylate) (PMMA), poly(2-hydroxyethyl methacrylate) (PHEMA), polystyrene (PS), and polydimethylsiloxane (PDMS), while the class of polyesters has been scarcely explored.

In this context, the present research was aimed at developing novel polymeric materials—more specifically, aromatic copolyesters—for potential use in the biomedical field. The reference homopolymer was poly(pentamethylene naphthalate) (PPeN), which was synthesized starting from a flexible glycolic moiety, 1,5-pentanediol, and rigid dimethyl 2,6-naphthalenedicarboxylate [33]. PPeN was selected due to its amorphous nature, its glass transition temperature above body temperature, and a very slow crystallization rate in a physiological environment [34]. This ensures high stability over time and limits the development of a crystalline phase, which could compromise the material’s transparency. PPeN was chemically modified by introducing 10 and 20 mol% of DMSI in its macromolecular chain. These relatively high molar fractions were deliberately chosen to maximize the surface wettability of the resulting materials without significantly compromising their molecular weight, thermal stability, or functional properties. Moreover, unlike, for example, aliphatic PEG-like moieties, aromatic DMSI serves as a rigid and hydrolytically stable co-unit, which does not increase chain flexibility, thereby mitigating the risk of a decrease in T_g_ towards 37 °C and the possible subsequent development of a crystalline phase. Based on these considerations, the homopolymer and the two copolymers, namely poly(pentamethylene naphthalate-co-sulfoisophthalate), P(PeN_x_PeSI_y_), where x and y represent the relative molar amounts of the two diesters, were synthesized using a solvent-free polycondensation approach. All the obtained materials were processed into thin films, and their molecular, structural, thermal, and mechanical properties were systematically investigated to establish structure–property relationships. Furthermore, surface wettability, water uptake, optical transparency, and preliminary in vitro biocompatibility assays were evaluated to explore the potential suitability of these materials for advanced functional applications in which optical properties are important, such as ocular ones.

## 2. Experimental Procedures

### 2.1. Materials

Dimethyl 2,6-naphthalenedicarboxylate (DMN, purity > 99%) was purchased from TCI Chemicals (Tokyo, Japan). Dimethyl 5-sulfoisophthalate sodium salt (DMSI, purity > 95%), 1,5-pentanediol (1,5-PeDO, purity > 97%), and titanium (IV) butoxide (TBT, purity > 97%) were provided by Sigma-Aldrich (St. Louis, MO, USA). All the chemicals were used as received from commercial suppliers without further purification.

### 2.2. P(PeN) and P(PeN_x_PeSI_y_) Syntheses

P(PeN) homopolymer and the two P(PeN_x_PeSI_y_) copolymers containing 10 and 20 mol% of polar comonomeric units were synthesized by a two-stage melt polycondensation process. During the first stage, the monomers, 1,5-PeDO and DMN in the case of the homopolymer, or 1,5-PeDO and the proper relative amounts of DMN and DMSI in the case of the copolymers, were introduced into a three-necked glass reactor together with the catalyst, TBT. In all cases, a 200 mol% excess of glycol with respect to the diester was employed. The first stage of the polymerization, lasting approximately 1.5 h, involved transesterification reactions, leading to the formation of oligomers, while methanol was continuously distilled off as a reaction by-product. This step was carried out at a constant temperature of 195 °C under a controlled nitrogen atmosphere and continuous mechanical stirring at 100 rpm. During the second stage, which lasted approximately 2 additional h, further transesterification reactions took place, leading to polymer chain growth. The reaction temperature was set at 230 °C while the pressure was progressively reduced to 0.075 mbar, thereby promoting the removal of excess glycol and methanol by distillation. Simultaneously, the torque value, which provides an indication of the viscosity of the polymer melt and, indirectly, of the polymer molecular weight, increased progressively. Once the torque had stabilized and no further distillation occurred, the polymer was discharged from the reactor.

### 2.3. Molecular Characterization

The chemical structure and copolymer composition were determined by ^1^H-NMR spectroscopy at room temperature using a Varian Inova 400 MHz (Agilent Technologies, Santa Clara, CA, USA) spectrometer. Samples were dissolved in deuterated chloroform (CDCl_3_) containing 0.03 vol% tetramethylsilane (TMS) at a concentration of 10 mg mL^−1^. A few drops of trifluoroacetic acid were added to this solution to promote the solubilization of the materials.

The intrinsic viscosity (η) was determined at 30 °C by means of a SCHOTT-Gerate (Mainz, Germany) UVS300 viscometer equipped with an electronic sensor for flow-time measurements and a Ubbelohde531 13/Ic capillary (internal diameter of 0.84 mm). Polymers were dissolved in phenol/1,1,2,2-tetrachloroethane (60/40% *w*/*w*) at four different concentrations (0.8, 0.7, 0.6, and 0.5 g/dL), and the measurements were repeated at least five times. The intrinsic viscosity (η) was obtained by extrapolating the linear plots of (ln η_r_)/C and η_sp_/C, where η_r_, η_sp_, and C are the relative viscosity, the specific viscosity, and the concentration, respectively.

### 2.4. Film Preparation

Polymer films with a thickness of approximately 50 μm were prepared by compression molding between two Teflon plates using a Carver (Wabash, IN, USA) C12 laboratory press. Each polymer was maintained at 300 °C for 2 min, and then a pressure of 10 ton m^−2^ was applied. Prior to characterization, the films were stored at room temperature for two weeks to ensure a uniform thermal history.

### 2.5. Thermal Characterization

Thermogravimetric analysis (TGA) was performed using a PerkinElmer (Waltham, MA, USA) TGA 4000 analyzer under a nitrogen atmosphere. Samples were heated from 40 to 800 °C at a constant heating rate of 10 °C min^−1^. The onset degradation temperature (T_onset_) was determined from the thermogravimetric curve, while the temperature corresponding to the maximum degradation rate (T_max_) was obtained from the first derivative of the weight-loss curve.

Differential scanning calorimetry (DSC) measurements were carried out on a PerkinElmer (Waltham, MA, USA) DSC 6 calorimeter under a nitrogen flow of 20 mL min^−1^. Samples (approximately 10 mg) were heated from 0 to 250 °C at 20 °C min^−1^ (first heating scan), held isothermally for 3 min, and subsequently rapidly cooled at 100 °C min^−1^. A second heating scan was then performed under the same conditions. The glass transition temperature (T_g_) was taken as the midpoint of the heat capacity step associated with the glass transition, and the corresponding heat capacity (Δc_p_) was determined from the step height.

### 2.6. Mechanical Characterization

Uniaxial tensile tests were carried out using an Instron 5966 universal testing machine (Norwood, MA, USA) equipped with pneumatic rubber-faced grips and a 1 kN load cell. Rectangular specimens (5 × 50 mm^2^) with a gauge length of 20 mm were tested at a crosshead speed of 10 mm min^−1^. Stress–strain curves were obtained from the recorded load–displacement data. The Young’s modulus (E) was calculated from the initial linear region of the stress–strain curve, whereas the stress at break (σ_b_) and elongation at break (ε_b_) were determined at the fracture point. For each material, at least five specimens were tested, and the results are reported as mean value ± standard deviation.

### 2.7. Wettability Measurements

Static water contact angle (WCA) measurements were performed at room temperature on compression-molded polymer films previously washed in a 70% *v*/*v* aqueous ethanol solution and then dried overnight at room temperature, using a DSA30S apparatus (Krüss Scientific, Hamburg, Germany). Droplets (4 μL) of deionized water were deposited onto the film surface, and their profiles were recorded immediately after deposition. Contact angles were determined by image analysis of these drops. For each sample, measurements were carried out at eight different locations, and the results are reported as the mean value ± standard deviation.

In order to qualitatively assess whether the polymers are capable of absorbing water, they were immersed in water for 24 h and subsequently analyzed by first-scan DSC under the conditions described above. Moreover, the water uptake capacity of the samples was quantitatively evaluated via an immersion test at predetermined time intervals ranging from 1 to 48 h. Prior to immersion, the samples were completely dried to a constant weight, and their dry weight (W_d_) was recorded. The specimens were then immersed in distilled water at room temperature (25 °C). At specific time points (1, 3, 6, 12, 24, and 48 h), the samples were withdrawn. Excess surface water was immediately removed with filter paper, and the wet weight (W_t_) was promptly recorded. The water uptake percentage was calculated according to the following equation:

Water Uptake % = [(W_t_ − W_d_)/W_d_] × 100

The results are reported as the mean value ± standard deviation of three different experiments.

Lastly, the presence of characteristic functional groups and the water incorporation were assessed by FT-IR analysis at room temperature, employing a PerkinElmer (Waltham, MA, USA) Spectrum 3 FT-IR Spectrometer.

### 2.8. Transparency and Color Evaluation

The color of the film samples was measured using a HunterLab ColorFlex EZ 45/0° color spectrophotometer (Reston, VA, USA) with D65 illuminant and a 10° observer according to ASTM E308 [35]. Measurements were recorded using the CIELAB scale. The instrument was calibrated using black-and-white tiles before the measurements. Results were expressed as L* (lightness), a* (red/green), and b* (yellow/blue) parameters. The total color difference (∆E) was calculated using the following equation:

∆E = [(∆L)^2^ + (∆a)^2^ + (∆b)^2^]^0.5^

where ∆L, ∆a, and ∆b are the differences between each sample color parameter (L*, a*, and b*) and the corresponding color parameter of a standard white plate used as the film background (L′ = 66.70, a′ = −0.76, and b′ = 0.40).

Chromaticity (C*) and hue angle (h_ab_) were calculated as previously reported in the literature [36,37,38,39], according to the following equations:

C* = [(a*)^2^ + (b*)^2^]^0.5^

h_ab_ = [arctan (b*/a*)/2π] 360

Measurements were recorded in triplicate at random positions over the film surface, and the results are reported as the average values ± standard deviation of these three measurements. Additionally, a quantitative assessment of the UV-Vis transmission properties was carried out by recording transmittance spectra of compression-molded films between 200 nm and 800 nm using a Varian Cary 100 (Agilent Technologies, Palo Alto, CA, USA) spectrophotometer.

### 2.9. In Vitro Preliminary Biocompatibility Tests

According to ISO 10993-5 guidelines [40], the preliminary biocompatibility of the investigated materials was assessed in vitro by means of the 3-(4,5-dimethylthiazol-2-yl)-2,5-diphenyltetrazolium bromide (MTT) assay. More specifically, cell viability was evaluated by measuring the metabolic activity of primary human keratocytes cultured in direct contact with the polymeric materials for up to five weeks. Primary human keratocytes were isolated from human donor corneal tissue obtained from Amnitrans EyeBank (Rotterdam, The Netherlands). Consent for the use of retrieved tissues for research purposes was obtained from the legally entitled families by Amnitrans EyeBank. The use of donated tissues was conducted in accordance with the Barcelona Principles [41].

Briefly, keratocytes were seeded in 96-well culture plates and incubated under standard culture conditions until sub-confluence was reached. In parallel, polymer films were cut into 2 × 2 mm^2^ specimens and sterilized by immersion in 90% ethanol for 30 min. The samples were allowed to dry under a sterile laminar flow hood and subsequently rinsed once with fresh culture medium before being placed in contact with the cells.

At each incubation time, cell viability was quantified using the MTT assay (Thiazolyl Blue Tetrazolium Bromide; cat. no. M5655; Sigma-Aldrich, St. Louis, MO, USA).

This quantitative colorimetric assay is based on the reduction of the yellow tetrazolium salt to insoluble purple formazan crystals by mitochondrial dehydrogenase enzymes in metabolically active cells. Accordingly, the amount of formazan produced is directly proportional to the number of viable cells. The resulting formazan crystals were dissolved in 2-propanol, and the absorbance was measured spectrophotometrically at 570 nm, with 650 nm used as the reference wavelength. Cell viability was expressed as the percentage of viable cells relative to the control, consisting of keratocytes cultured under standard conditions in culture medium without polymeric materials. Each data point represents the mean of two independent experiments, each performed in quintuplicate. Statistical analyses were performed using GraphPad Prism (version 10.2.1; GraphPad Software, San Diego, CA, USA). For biological assays, a minimum of four technical replicates were performed for each experimental condition within each independent biological experiment. Technical replicates were averaged and treated as a single biological observation. Time-course MTT data were analyzed using a two-way repeated-measures ANOVA, followed by Dunnett’s multiple-comparison test to compare each tested material with the control at each time point. Statistical significance was set at *p* < 0.05, and 95% confidence intervals were calculated for multiple comparisons. Formal tests of normality were not performed because only two independent biological replicates were available, which was considered insufficient for a meaningful assessment of data distribution. No formal sample randomization or experimental blinding was performed. Samples were assigned to predefined experimental groups according to the experimental design and processed under identical experimental conditions, and the experimental groups were known to the investigators during both experimental procedures and data analysis.

## 3. Results and Discussion

### 3.1. Synthesis, Processing, and Molecular Properties

**Figure 1:**
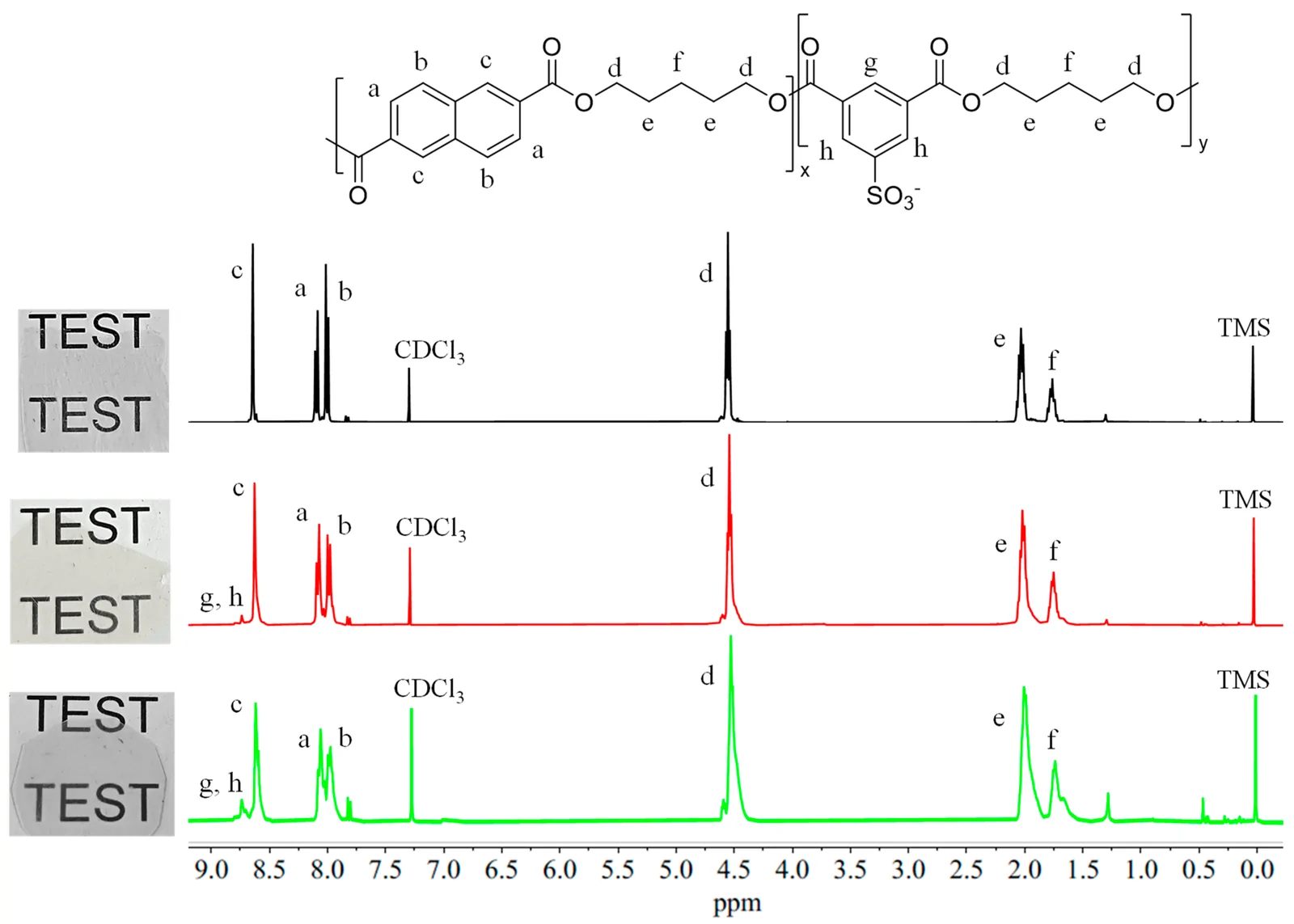
^1^H-NMR spectra of P(PeN) (black), P(PeN_90_PeSI_10_) (red), and P(PeN_80_PeSI_20_) (green) with the corresponding signal assignment, together with pictures of the transparent compression-molded films.

Poly(pentamethylene naphthalate) (PPeN) homopolymer and its copolyesters, containing 10 and 20 mol% of pentamethylene sulfoisophthalate co-units, were successfully synthesized by melt polycondensation and processed into thin compression-molded films. At room temperature, they all appear as transparent, nearly colorless solids (Figure 1).

In order to confirm the chemical structure and composition of the synthesized polymers, ^1^H-NMR spectroscopy was carried out. All spectra, shown in Figure 1, were consistent with the expected structures, demonstrating good control over the polymerization process. More specifically, in all cases, in addition to the peaks of the solvent (CDCl_3_, δ 7.25 ppm) and the reference (TMS, δ 0 ppm), only signals attributable to the protons of the polymers are present. In more detail, as regards the PPeN homopolymer, the naphthalene ring shows two doublets at δ 8.02 ppm (a, 2H) and δ 7.93 ppm (b, 2H), respectively, and a singlet at δ 8.67 ppm (c, 2H). For the aliphatic glycol moiety, the signals of protons in the α-position to the ester oxygen are evident at δ 4.51 ppm (d, triplet, 4H), while those of inner protons are located at δ 1.96 ppm (e, multiplet, 4H) and δ 1.74 ppm (f, multiplet, 2H), respectively. If the two copolymers are considered, in addition to the aforementioned peaks, the partially overlapped signals due to the protons of the sulfoisophthalate ring are located at δ 8.75 ppm (g, h, 3H). The actual molar composition was obtained from the relative ratio between the normalized intensities of the c peak, referring to the naphthalene ring, and the g and h peaks for the sulfoisophthalate subunit. As can be seen from the data in Table 1, the effective compositions were close to the feed ones, confirming the good control over the synthetic process.

Viscometric analyses in dilute solution (I.V.) were carried out for all samples to obtain an indirect estimation of the molecular weight of the synthesized materials. The PPeN homopolymer exhibited a relatively high intrinsic viscosity, in line with literature data [34], which decreased as the co-unit content increased. This behavior, already observed for other polymeric systems containing sulfoisophthalate moieties [42,43], may be due to the high hydrodynamic bulkiness of the comonomeric unit, which introduces steric hindrance [43]. Moreover, the pendant polar sulfonate groups promote the formation of intermolecular interactions, which further limit polymer chain mobility and ultimately hinder the growth of the macromolecules. However, all the materials discharged from the reactor were found to be easily processable as thin, manageable films, a fundamental requirement for their possible applicability.

### 3.2. Thermal Properties

**Figure 2:**
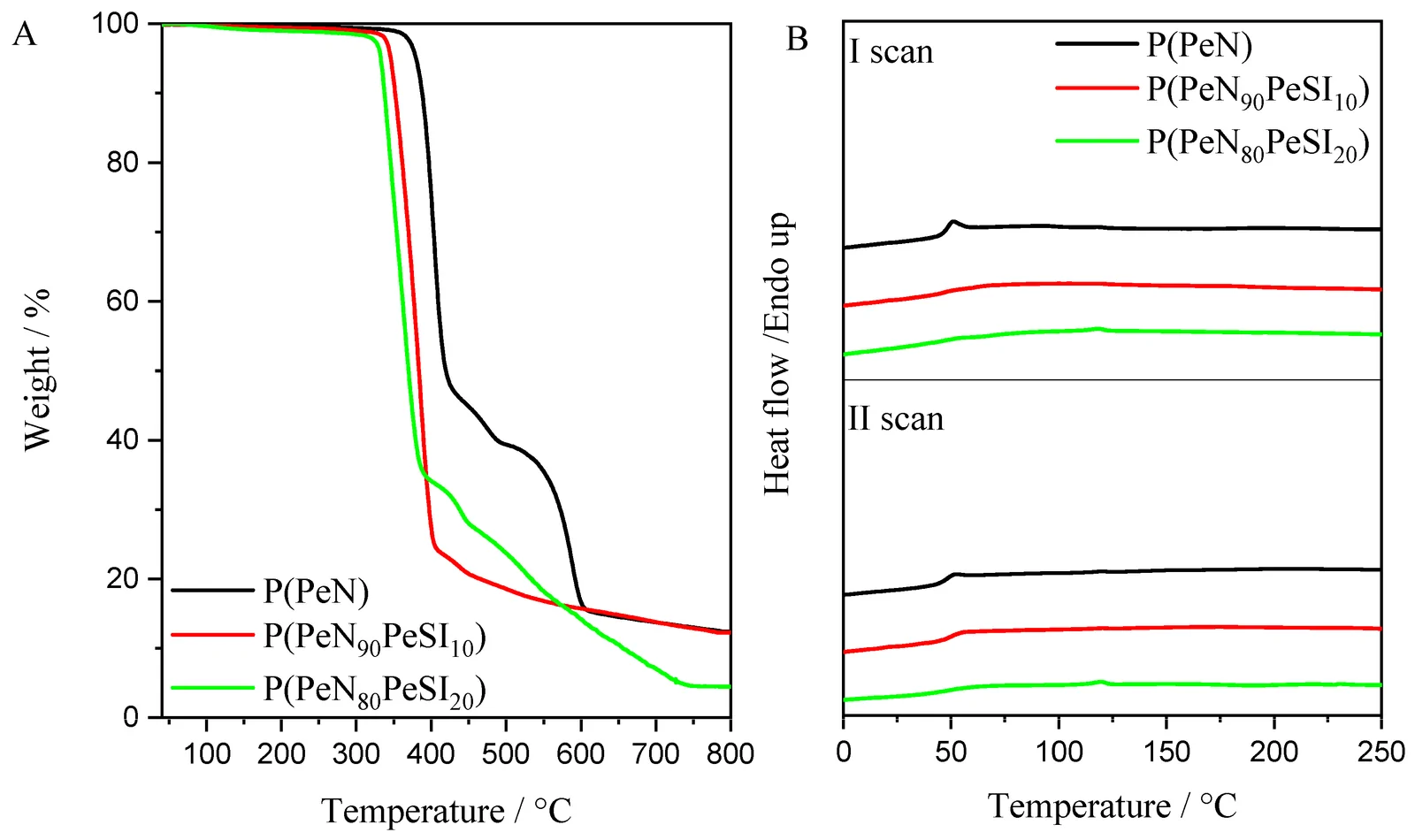
(**A**) TGA traces and (**B**) calorimetric curves (I and II scans) of PPeN and P(PeN_x_PeSI_y_) copolymers.

To assess the thermal stability of the materials under study, TGA analysis was carried out. The obtained thermograms are shown in Figure 2A, while the temperatures of the onset of degradation (T_id_) and maximum weight loss rate (T_max_) are listed in Table 1.

All three materials are characterized by high thermal stability, with a T_id_ of 330 °C or higher and T_max_ greater than 360 °C, temperatures that allow for a wide processability window. These values align with those reported for other sulfonated terephthalate- and furan-based copolymers (Table S1) [21,22,23], considering that their intrinsic viscosities were slightly higher than those of the materials under study. In detail, the homopolymer proved to be the material with the highest thermal stability, with a T_id_ of 386 °C and T_max_ equal to 407 °C. A progressive decrease in these temperatures can then be noted, correlated with the increase in the polar group content, indicating a slight deterioration in thermal stability. This trend is probably caused by the decomposition of more reactive functional groups within the polar co-unit [25]. Furthermore, all the curves (Figure 2A) show multiple degradation steps. This behavior is more evident for PPeN, probably due to the high viscosity of this sample, which likely hinders the diffusion of volatile degradation products from the polymer melt during heating. The weight loss observed at the end of the test was not complete in all three cases, with a residual char slightly lower than 20% for the homopolymer and P(PeN_90_PeSI_10_), while for the copolymer richest in PeSI co-unit, the residual char is only about 5%, confirming the lower stability of this polar moiety compared to the naphthalene ring under an inert atmosphere.

The three polymeric films were then characterized from a calorimetric point of view to obtain information about their main thermal transitions and their crystallization capability. The I and II scan DSC traces are shown in Figure 2B, while the associated thermal data are listed in Table 1. As observed from the first heating scans, all the films, after storage at room temperature for two weeks, exhibited the typical behavior of amorphous polyesters, with the calorimetric curves showing only an endothermic deviation of the baseline, attributable to the glass transition, above room temperature. In detail, PPeN showed a T_g_ of 46 °C, in line with literature data [16,17], while copolymerization resulted in a gradual and slight increase in T_g_, proportional to the amount of co-unit (Table 1), reaching values of 50 °C for P(PeN_90_PeSI_10_) and 52 °C for P(PeN_80_PeSI_20_). The expected progressive reduction in macromolecular mobility with increasing sulfonate group content can be attributed to the combined effect of steric hindrance [43] and intermolecular interactions [25,44], which limit chain mobility, as already observed in the literature [22,23]. After the first scan, a second scan was performed, preceded by rapid cooling from 250 °C. As expected, the obtained curves are very similar to those of the first scan (Figure 2B, Table 1).

Notably, all three materials exhibited glass transition temperatures above the physiological temperature (37 °C), indicating that they remain in the glassy state under in vivo conditions. This prevents thermally induced changes in their physical properties, particularly crystallization, which would compromise their optical transparency. Furthermore, the absence of crystallization and melting transitions in the DSC thermograms confirms the fully amorphous nature of the copolyesters, enabling the preparation of highly transparent films. Collectively, these characteristics make the synthesized materials promising candidates for biomedical applications, particularly when optical transparency is a fundamental requirement.

### 3.3. Evaluation of Wettability

**Figure 3:**
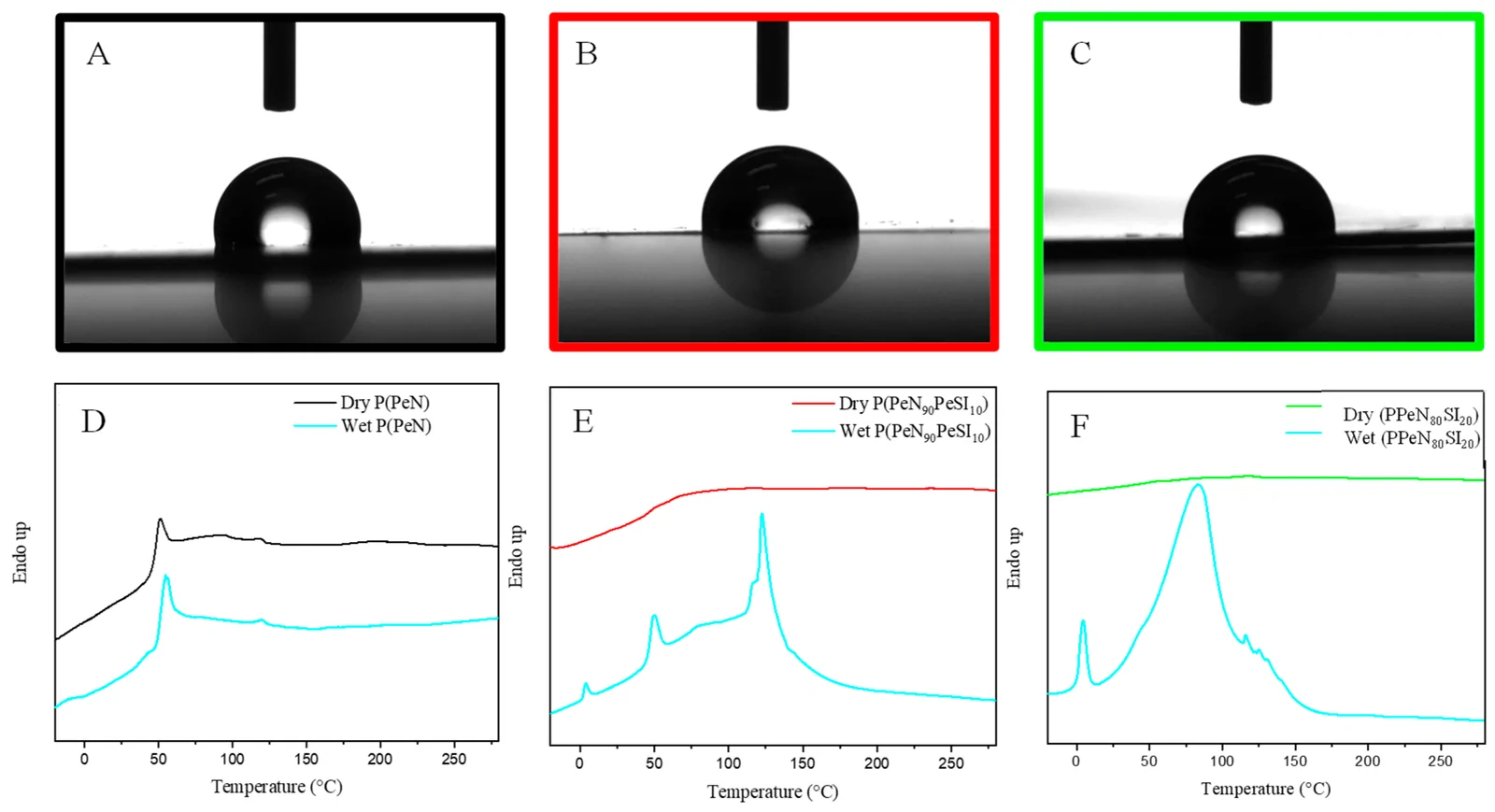
Water drops on film surfaces of (**A**) PPeN, (**B**) P(PeN_90_PeSI_10_), and (**C**) P(PeN_80_PeSI_20_) copolymers. Calorimetric curves (I scan) of (**D**) PPeN, (**E**) P(PeN_90_PeSI_10_), and (**F**) P(PeN_80_PeSI_20_) copolymers before and after immersion in water for 24 h.

The compression-molded films were subjected to water contact angle (WCA) measurements under static conditions to assess the hydrophilicity/hydrophobicity of their surfaces. The contact angle values obtained are listed in Table 1, while pictures of the droplets deposited on the surface of each film are shown in Figure 3A–C. The homopolymer exhibits hydrophobic behavior (WCA > 90°), while the copolymers, as expected, show progressively lower WCA values, all below 90°, proportional to the amount of co-unit, indicating greater hydrophilicity than the homopolymer. This behavior is due, once again, to the presence of pendant sulfonate groups, which are highly polar and strongly interact with water molecules, increasing the overall hydrophilicity [45,46,47].

To further determine whether the investigated copolymers are capable of interacting with water more efficiently than the reference homopolymer, they were immersed in water for 24 h, and the DSC analysis was performed before and after immersion. As can be seen in Figure 3D–F, while the I scan traces of the homopolymer are practically identical, substantial differences can be observed in the copolymers. In fact, in both cases, a very intense endothermic peak centered around 100 °C, attributable to the evaporation of water absorbed by each sample, appeared. Furthermore, the intensity of this peak is qualitatively proportional to the amount of co-unit, confirming that copolymerization likely improved water uptake.

Subsequently, to quantify the actual water uptake of the samples, immersion tests were also carried out. As shown in Figure S1A, PPeN homopolymer showed no appreciable weight change during the entire experiment. A similar trend was observed for P(PeN_90_PeSI_10_), which exhibited a water uptake percentage of around 1% over the investigated period. Conversely, the copolymer richest in DMSI co-units showed a progressive mass increase that was directly correlated with immersion time, reaching a value above 60% after 48 h of immersion (Figure S1A). This finding further supports the previously performed WCA and DSC analyses (Figure 3). It should be pointed out that although this high water uptake is an advantageous feature for many biomedical applications, it may compromise dimensional stability and mechanical performance via swelling and plasticization. Therefore, further evaluations will be carried out to better address these aspects.

Finally, the samples subjected to water uptake tests were characterized by FT-IR measurements to evaluate any differences in the collected spectra after different immersion times. As shown in Figure S1B–D, all samples exhibited the characteristic functional group absorption bands of naphthalate polyesters [48]. These include the naphthalene ring vibration at approximately 800 cm^−1^, the ester carbonyl (C=O) stretching vibration at around 1720 cm^−1^, the asymmetric C–O–C stretching vibration near 1250 cm^−1^, and the aliphatic C–H_2_ stretching vibrations at 2920 and 2850 cm^−1^. Additionally, the copolymer spectra displayed a bending vibration peak at 630–620 cm^−1^ and a stretching vibration peak around 1050 cm^−1^, both corresponding to the sulfonate groups [49]. Following immersion in water, a broad O–H stretching band emerged between 3400 and 3500 cm^−1^, accompanied by a smaller H–O–H bending vibration at 1600–1640 cm^−1^ within the copolymers. These bands were more pronounced in the composition with the highest DMSI content and became progressively more intense with increasing immersion time. Conversely, the spectra of the homopolymer remained practically unaltered after water immersion, in line with previous analyses.

### 3.4. Mechanical Properties

**Figure 4:**
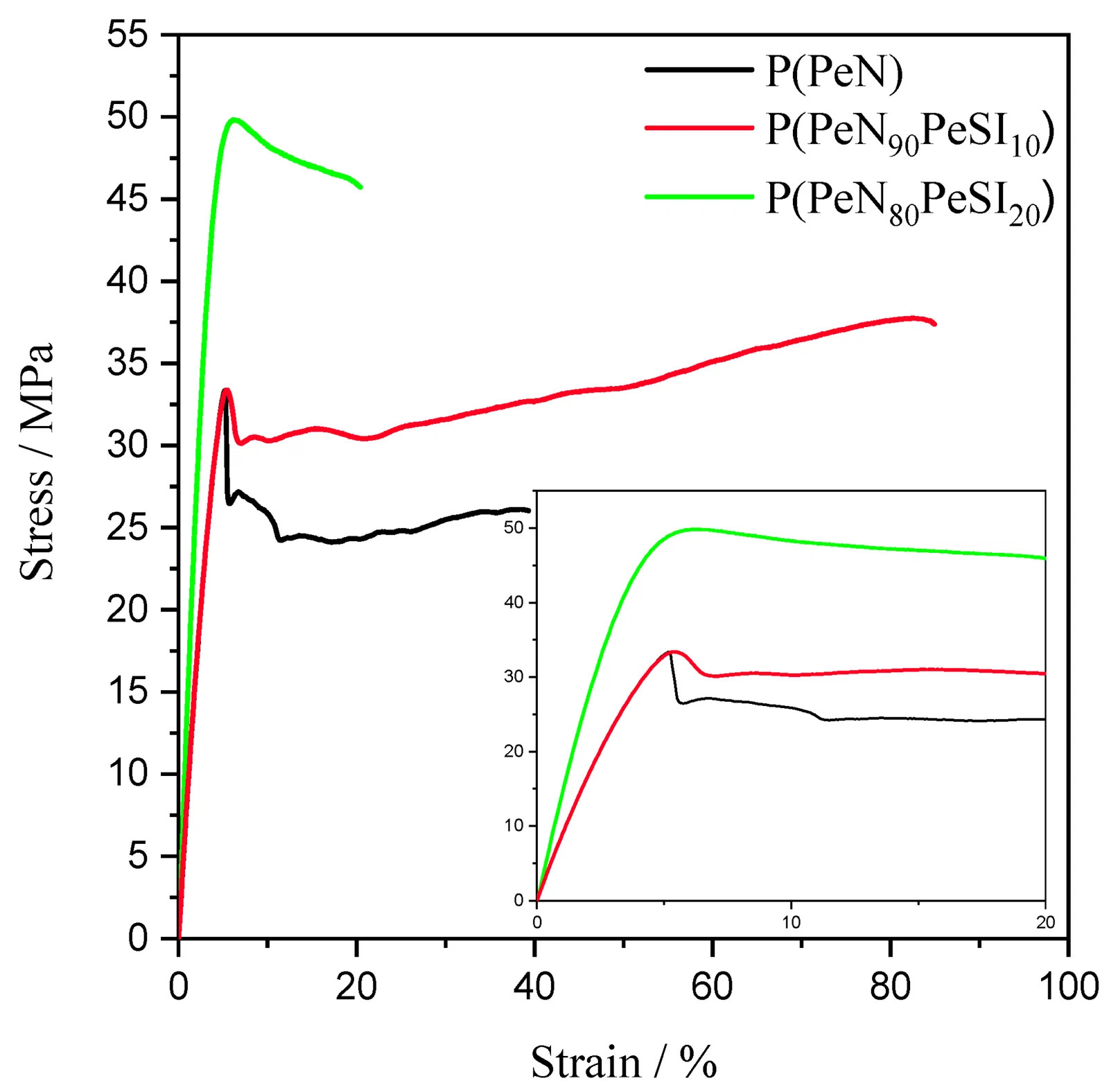
Representative stress–strain curves of PPeN and P(PeN_x_PeSI_y_) copolymers. In the inset, an enlargement of the region corresponding to low strain values (up to 20%) is shown.

In order to obtain information on the mechanical properties of the synthesized materials, tensile tests were conducted on PPeN and its copolymers in the form of films. The stress–strain curve of the homopolymer shows the behavior typical of a brittle material, characterized by an elastic modulus of approximately 900 MPa, a tensile strength of approximately 25 MPa, and a limited elongation at break, around 50% (Table 2, Figure 4). This behavior, which is not surprising considering that the material is glassy at room temperature (Table 1), is also in line with literature data [16,17]. As to the copolymers, the effect of copolymerization results in a progressive increase in the elastic modulus, which reaches approximately 1500 MPa for the copolymer containing the highest amount of co-unit, and in the tensile strength, which increases from 25 MPa for PPeN to 45 MPa for P(PeN_80_PeSI_20_). This behavior, which is in line with other copolymeric systems containing sulfonate co-units (Table S1), is due to the insertion of a rigid and polar co-unit within the homopolymer chain, as confirmed by the progressive increase in T_g_ (Table 1). Regarding elongation at break, the P(PeN_90_PeSI_10_) copolymer exhibited the most ductile behavior within the series (Table 2), with an ε_B_ value reaching 80%. Conversely, P(PeN_80_PeSI_20_) shows the lowest elongation at break, below 20%, which resulted in a decrease in ductility compared to the other copolymeric composition.

In order to explain this trend, it should be noted that the observed mechanical behavior reflects the two competing effects of molecular weight and intermolecular interactions. Since copolymerization leads to a decrease in molecular weight (Table 1), which is known to strongly influence elongation at break, the poor ductility of P(PeN_80_PeSI_20_) is expected despite its higher concentration of sulfonate co-units capable of promoting intermolecular interactions. Conversely, in P(PeN_90_PeSI_10_), the enhancement in interchain interactions outweighs the modest reduction in molecular weight, leading to an overall improvement in elongation at break. A further hypothesis, used to support similar results obtained for terephthalate-based copolymers containing similar molar amounts of DMSI [23] (Table S1), is that low contents of this co-unit may form small aggregates, which could act as reversible chain extenders, thus increasing copolymer ductility. Conversely, at higher contents, these aggregates may behave as defects, decreasing the deformation at break.

### 3.5. Color and Optical Appearance

The optical appearance of PPeN and its copolymers was evaluated using the CIE Lab parameters L*, a*, and b*, along with the resulting total color difference (ΔE), chroma (C*), and hue angle (h_ab_), as reported in Table 3.

The most pronounced variation as a function of composition was observed for the lightness parameter. In detail, PPeN showed a very low L* value (2.06 ± 0.12), which increased only slightly with the incorporation of 10 mol% PeSI (Table 3). In contrast, a considerably higher and significantly different (*p* < 0.05) L* value was recorded for P(PeN_80_PeSI_20_). Since L* describes lightness rather than light transmission itself, this change should not be directly interpreted as a quantitative variation of transparency. As to chromaticity coordinates, they showed relatively minor changes, with a* values close to zero for all films analyzed (Table 3). Although statistically significant differences were found between the compositions, the small absolute magnitude of these values indicates only a weak contribution along the red-green axis. Similarly, negative b* values were obtained for all samples, with no statistically significant differences, indicating that the blue component of the films remained essentially unchanged by varying the copolymer composition. The hue angle values were consistent with the negative b* coordinates (Table 3). These values were also all close to 270°, corresponding to the blue region of the color wheel, and therefore indicate a weak bluish component of the films. Although statistically significant differences were found between the three h_ab_ values, the relatively narrow angular range indicates that increasing the PeSI fraction did not cause a substantial shift toward a different hue region. Chromaticity (C*) values, related to color saturation, were low and similar for all compositions, suggesting that none of the studied compositions developed a pronounced intrinsic coloration. A clear composition-dependent trend was also observed for the total color difference, ΔE, calculated relative to the reference white. Indeed, PPeN and P(PeN_90_PeSI_10_) showed similar values, 64.70 ± 0.12 and 63.22 ± 0.83, respectively, while ΔE decreased significantly to 42.43 ± 0.82 for P(PeN_80_PeSI_20_). Considering that this sample simultaneously showed a marked increase in L* while maintaining a low C* value, the decrease in ΔE may be primarily associated with the change in lightness compared to the reference white rather than the development of a more saturated color.

Overall, the results indicate that the incorporation of PeSI predominantly affects the films’ lightness and, consequently, their overall optical appearance, while its influence on color saturation and hue is relatively limited. In particular, the optical characteristics of P(PeN_90_PeSI_10_) remain similar to those of PPeN, while further increasing the PeSI content results in a marked increase in lightness. However, all the films analyzed maintain low chromaticity values and only a weak bluish hue.

To quantitatively assess film transparency, UV-Vis measurements were carried out. According to the obtained spectra (Figure S2), the PPeN homopolymer exhibits a transmittance close to 80% at 550 nm, which provides an estimate of film transparency [50]; this value decreases only slightly for the P(PeN_90_PeSI_10_) copolymer. Notably, these transmittance values are comparable to, or even in line with, those of other polymeric materials investigated for biomedical applications, including but not limited to ocular devices [51,52,53]. Conversely, a substantial decrease in transmittance, down to approximately 50%, was observed for the film with the highest PeSI content. These results are particularly relevant considering they were obtained from relatively thick films (around 50 μm). Consequently, transparency could be further enhanced by reducing the film thickness.

### 3.6. Preliminary in Vitro Biocompatibility Evaluation

**Figure 5:**
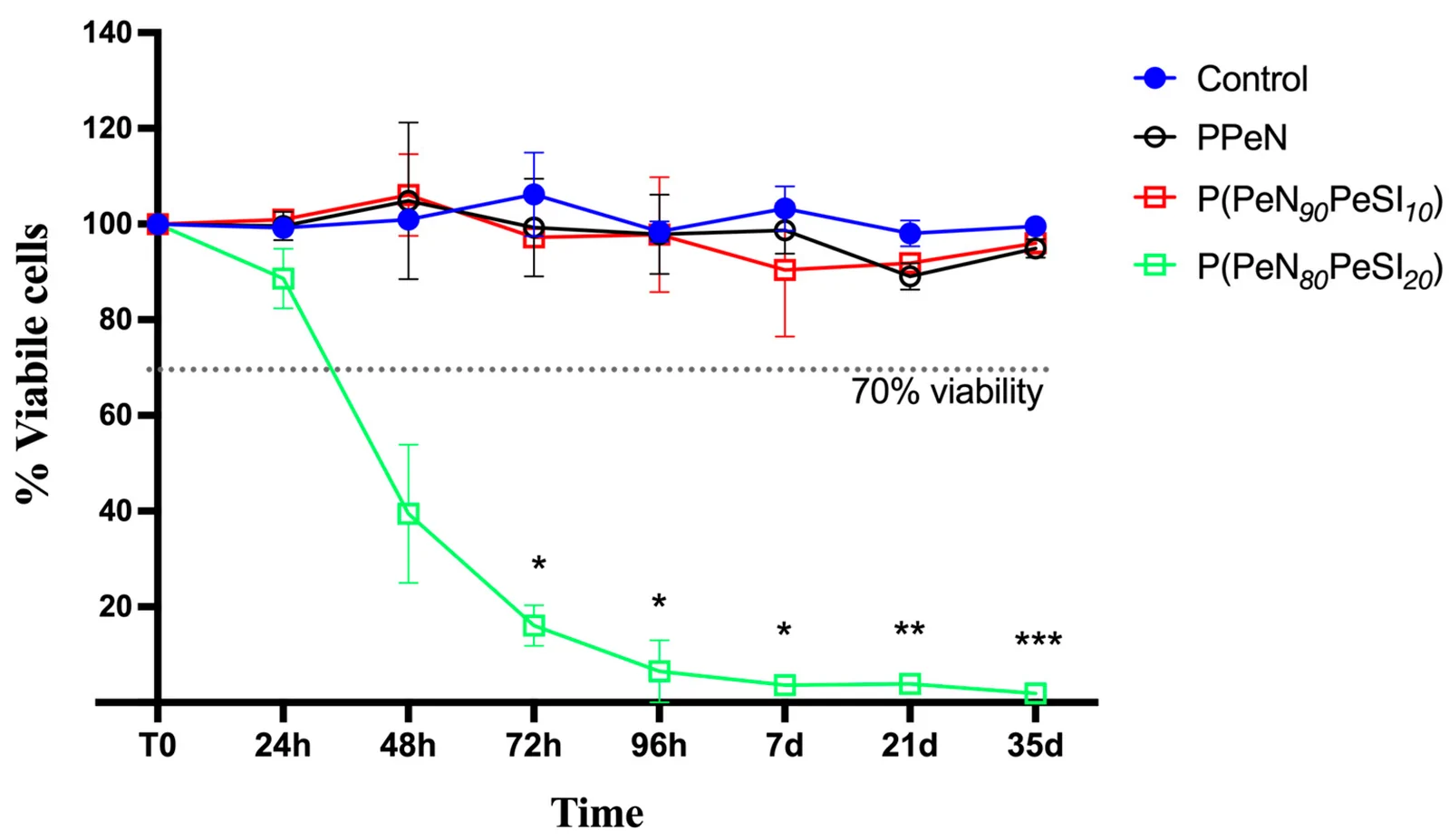
Cell viability data for PPeN and P(PeN*_x_*PeSI*_y_*) copolymers compared to the control. Two-way repeated-measures ANOVA, * *p* < 0.05; ** *p* < 0.01; *** *p* < 0.0001.

The in vitro biocompatibility of the synthesized materials was preliminarily evaluated by means of the MTT assay. For this purpose, primary human keratocytes, isolated from human corneal stroma, were cultured in direct contact with the different polymeric materials for five weeks. Cell viability was expressed as a percentage relative to the control (CTR), consisting of cells cultured in the absence of any material. The corresponding cell viability data are presented in Figure 5.

As shown in the figure, both the homopolymer and P(PeN_90_PeSI_10_) copolymer maintained excellent cell viability (approximately 100%) throughout the entire incubation period, exhibiting a behavior comparable to that of the control. In contrast, P(PeN_80_PeSI_20_) showed a progressive decrease in cell viability over time, becoming cytotoxic (cell viability < 70%) after only 48 h of incubation. These findings indicate that human corneal stromal cells can maintain normal viability only in the presence of low concentrations of polar groups within the polymer backbone, whereas a higher concentration appears to impair cellular metabolic activity, likely owing to unfavorable interactions with the cell membrane and/or the extracellular microenvironment. Therefore, optimizing the copolymer composition is crucial to ensure the safety of these materials for potential biomedical applications.

## 4. Conclusions

The biomedical field requires increasingly advanced materials capable of meeting stringent requirements in terms of biocompatibility, mechanical strength, flexibility, and hydrophilicity. Among the various classes of biomaterials, polymers, and particularly polyesters, represent a highly promising solution owing to their broad range of physicochemical properties, ease of processing, and the possibility of chemical modification and functionalization to optimize their interactions with the biological environment. For these reasons, the present research focused on the synthesis of novel random copolyesters of poly(pentamethylene naphthalate) containing different molar amounts of polar sulfoisophthalate moieties (10 and 20%, respectively), to obtain an attractive polymeric system potentially suitable for biomedical applications. The solvent-free copolymerization approach mitigated some limitations of the reference homopolymer by increasing its hydrophilicity and water uptake while preserving its desirable properties, including high thermal stability. Overall, PeSI incorporation modified the optical appearance of PPeN-based films primarily through a decrease in optical transmittance and lightness changes, which became particularly noticeable at 20 mol% of PeSI, while preserving low chromaticity and a faint bluish hue without introducing pronounced intrinsic coloration. Moreover, according to a preliminary in vitro biocompatibility evaluation, the material containing the lowest amount of comonomeric unit maintained cell viability, suggesting it as a promising candidate for the intended application, due to a favorable balance among the required properties (such as improved surface hydrophilicity and mechanical performance, together with high thermal stability, proper optical transparency, and cell viability). Conversely, increasing the comonomer content resulted in enhanced hydrophilicity but was accompanied by a progressive deterioration of both the mechanical ductility and cell viability. Overall, although these findings are preliminary and require further validation, this study paves the way for new applications of naphthalene-based polymers in the biomedical field, particularly where transparency is of primary importance.

## Figures and Tables

**Table 1:** Molecular (^1^H-NMR, I.V.), thermal (TGA, I scan and II scan DSC), and surface wettability (WCA) characterization data of PPeN and P(PeN_x_PeSI_y_) copolymers.

	^1^H-NMR		I.V.	TGA		I SCAN DSC		II SCAN DSC		WCA
	PeN Feed	PeN Real	η	T_id_	T_max_	T_g_	Δc_p_	T_g_	Δc_p_	θ
	mol%	mol%	dL/g	°C	°C	°C	J/g°C	°C	J/g°C	°
P(PeN)	100	100	0.53	386	407	46	0.200	46	0.202	95 ± 5
P(PeN_90_PeSI_10_)	90	92	0.41	354	386	50	0.173	50	0.309	86 ± 4
P(PeN_80_PeSI_20_)	80	85	0.25	330	362	52	0.088	52	0.116	81 ± 5

**Table 2:** Mechanical characterization data (elastic modulus (E), tensile stress (σ_B_), and percentage elongation at break (ε_B_) obtained from stress–strain curves) of PPeN and P(PeN_x_PeSI_y_) copolymers.

	P(PeN)	P(PeN_90_PeSI_10_)	P(PeN_80_PeSI_20_)
E/MPa	807 ± 24	878 ± 57	1315 ± 84
σ_B_/MPa	22 ± 2	39 ± 4	41 ± 1
ε_B_/%	44 ± 8	82 ± 30	17 ± 5

**Table 3:** Lightness coefficient (L*), a* and b*, total color difference (ΔE), C*, and h_ab_ of PPeN and P(PeN_x_PeSI_y_) copolymers.

Sample	L*	a*	b*	ΔE	C*	h_ab_
White standard	66.70 ± 0.03	−0.76 ± 0.01	0.40 ± 0.01	-	0.86	152.44 ± 0.44
PPeN	2.06 ± 0.12 ^b^	0.35 ± 0.02 ^a^	−2.13 ± 0.07 ^a^	64.70 ± 0.12 ^a^	2.16 ± 0.07 ^a^	279.32 ± 0.58 ^a^
P(PeN_90_PeSI_10_)	3.53 ± 0.85 ^b^	−0.03 ± 0.03 ^c^	−1.83 ± 0.58 ^a^	63.22 ± 0.83 ^a^	1.83 ± 0.58 ^a^	268.93 ± 1.46 ^c^
P(PeN_80_PeSI_20_)	24.38 ± 0.81 ^a^	0.10 ± 0.03 ^b^	−2.56 ± 0.21 ^a^	42.43 ± 0.82 ^b^	2.56 ± 0.21 ^a^	272.17 ± 0.57 ^b^

h_ab_ = 0°, red-purple; h_ab_ = 90°, yellow; h_ab_ = 180°, green; h_ab_ = 270°, blue. Different lowercase letters within the same column indicate statistically significant differences between samples (*p* < 0.05), based on ANOVA, followed by Tukey’s post hoc test. The white standard was used exclusively as the reference background and was not included in the statistical comparison among film samples.

## Data Availability

Raw data are available from the corresponding authors upon reasonable request.

## Supplementary Materials

The following supporting information can be downloaded at https://www.mdpi.com/article/10.3390/polym18182212/s1, Table S1. Molecular (^1^H-NMR, I.V.), thermal (TGA, I scan DSC), and mechanical (stress–strain measurements) characterization data of PPeN and P(PeN_x_PeSI_y_) copolymers compared to those of other aromatic systems reported in the literature containing DMSI as a co-unit. Figure S1. (A) Quantitative water absorption data of PPeN and P(PeN_x_PeSI_y_) copolymers at different time points (1–48 h); FT-IR spectra before and after water immersion at different time points (1–48 h) of (B) PPeN, (C) P(PeN_90_PeSI_10_), and (D) P(PeN_80_PeSI_20_). Figure S2. UV–vis light transmission spectra of PPeN and P(PeN_x_PeSI_y_) copolymer films (thickness of ~50 μm).

## References

[1] Noor S., Sayed Daud S., Sudirman R., Noorul M., Mohd Norddin A., Jaafar J., Mahmood N.H. (2021). Applications of Synthetic Aromatic Polymers in the Biomedical Field: A Review. Malays. J. Med. Health Sci..

[2] Ye H., Zhang K., Kai D., Li Z., Loh X.J. (2018). Polyester Elastomers for Soft Tissue Engineering. Chem. Soc. Rev..

[3] Zhao Y., Zhong W. (2023). Recent Progress in Advanced Polyester Elastomers for Tissue Engineering and Bioelectronics. Molecules.

[4] Sreeja S., Muraleedharan C.V., Varma P.R.H., Sailaja G.S. (2020). Surface-Transformed Osteoinductive Polyethylene Terephthalate Scaffold as a Dual System for Bone Tissue Regeneration with Localized Antibiotic Delivery. Mater. Sci. Eng. C.

[5] Sughanthy S.A.P., Ansari M.N.M., Atiqah A. (2020). Dynamic Mechanical Analysis of Polyethylene Terephthalate/Hydroxyapatite Biocomposites for Tissue Engineering Applications. J. Mater. Res. Technol..

[6] Kim S.J. (2025). Polyethylene Terephthalate as an Artificial Ligament and Its Various Medical Applications. Ligament Reconstruction for Spine.

[7] Liguori A., De Vita A., Rossi G., Dolci L.S., Panzavolta S., Gualandi C., Mercatali L., Ibrahim T., Focarete M.L. (2022). A Modular Composite Device of Poly(Ethylene Oxide)/Poly(Butylene Terephthalate) (PEOT/PBT) Nanofibers and Gelatin as a Dual Drug Delivery System for Local Therapy of Soft Tissue Tumors. Int. J. Mol. Sci..

[8] Das A., Chethan K.N., Salins S.S., Shetty R., Shetty S. (2025). Poly (Butylene Adipate-Co-Terephthalate) (PBAT) in Biomedical Applications: A Comprehensive Review of Material Properties, Fabrication Methods, and Biofunctional Potential. Mater. Res. Express.

[9] Bondi E., Restivo E., Soccio M., Guidotti G., Bloise N., Motta I., Gazzano M., Ruggeri M., Fassina L., Visai L. (2025). Design and Characterization of Aromatic Copolyesters Containing Furan and Isophthalic Rings with Suitable Properties for Vascular Tissue Engineering. Int. J. Mol. Sci..

[10] Bondi E., Obino G., Guidotti G., Sensini A., Nagy M., Spronk H.M.H., van Griensven M., Lepedda A.J., Lotti N., Moroni L. (2026). Aromatic Copolyesters Based on Poly(Butylene Furanoate) and Poly(Butylene Isophthalate) for Small-Diameter Vascular Applications. ACS Biomater. Sci. Eng..

[11] Reczkowski J., Pruszkowska M.M., Jakubowski M., Ratajczak M., Ławniczak Ł., Szymkuć W., Chodkowski M., Antos-Bielska M., Krzyżowska M., Spriano S. (2025). Polyester artificial ligament modified with a polyphenol-zinc layer for controlled and targeted release of ciprofloxacin. Appl. Surf. Sci..

[12] Juma H., Zhao C., Wang Q., Guo Y., Fan X., Fan W., Zhao L., Sun J., Wang D., Wang Y. (2025). Enhanced Antioxidant and Antibacterial Properties of Polybutylene Adipate-Terephthalate/Curcumin Composite Films Using Surface-Modified Cellulose Nanocrystals. Polymers.

[13] Khan M.Z., Taghavian H., Wiener J., Militky J., Wang Y., Tomkova B., Cernik M., Dvorak L. (2024). Green in-situ immobilization of ZnO nanoparticles for functionalization of polyester fabrics. Surf. Interfaces.

[14] Ulery B.D., Nair L.S., Laurencin C.T. (2011). Biomedical Applications of Biodegradable Polymers. J. Polym. Sci. B Polym. Phys..

[15] Yamanobe T., Matsuda H., Imai K., Hirata A., Mori S., Komoto T. (1996). Structure and Physical Properties of Naphthalene Containing Polyesters I. Structure of Poly(Butylene 2,6-Naphthalate) and Poly(Ethylene 2,6-Naphthalate) as Studied by Solid State NMR Spectroscopy. Polym. J..

[16] Lee S.C., Chae K.Y. (2012). Miscibility with Thermal and Mechanical Properties for Poly(Pentamethylene 2,6-Naphthalate) and Poly(Heptamethylene 2,6-Naphthalate) Blends. Fibers Polym..

[17] Jeong Y.G., Jo W.H., Lee S.C. (2004). The Effect of Flexible Chain Length on Thermal and Mechanical Properties of Poly(m-Methylene 2,6-Naphthalate)s. Polymer.

[18] Li J., Liu C., Su K., Li Z. (2025). A Novel Poly (Pentylene 2,5-Furandicarboxylate-Co-Pentylene 2,6-Naphthalate) Polyester Derived from Bio-Based 2,5-Furandicarboxylic Acid with Superior Gas Barrier Properties. Food Chem..

[19] Global 2,6-Naphthalene Dicarboxylic Acid Market Insights—Industry Share, Sales Projections, and Demand Outlook 2026–2032. https://qyresearch.in/report-details/3915082.

[20] 6-Naphthalene Dicarboxylic Acid Market: $259.5M, 1.7% CAGR. https://www.datainsightsmarket.com/reports/26-naphthalene-dicarboxylic-acid-1114734.

[21] Gaona O., Kint D.P.R., De Ilarduya A.M., Alla A., Bou J., Muñoz-Guerra S. (2003). Preparation and Hydrolytic Degradation of Sulfonated Poly(Ethylene Terephthalate) Copolymers. Polymer.

[22] Zhang J., Li Z., Wu L. (2025). Synthesis and Antistatic Property of Poly(Ethylene 2,5-Furandicarboxylate) (PEF) and Sulfonated PEF. Polymer.

[23] Bautista M., Martínez De Ilarduya A., Alla A., Muñoz-Guerra S. (2013). Sulfonated Poly(Hexamethylene Terephthalate) Copolyesters: Enhanced Thermal and Mechanical Properties. J. Appl. Polym. Sci..

[24] Yoo Y.T., Lee B.J., Han S.I., Im S.S., Kim D.K. (2003). Physical Properties and Biodegradation of Poly(Butylene Adipate) Ionomers. Polym. Degrad. Stab..

[25] Zhou T., Wang X., Liu Y., Zheng L. (2023). A Green and Efficient Synthetic Strategy for the Preparation of PBS Ionomers with High Molecular Weight, High Ionic Group Content and Good Combined Properties. Chem. Eng. J..

[26] Lv L., Wu F., Chen S.C., Wang Y.Z., Zeng J.B. (2015). Properties Regulation of Poly(Butylene Succinate) Ionomers through Their Ionic Group Distribution. Polymer.

[27] Han S.I., Kang S.W., Kim B.S., Im S.S. (2005). A Novel Polymeric Ionomer as a Potential Biomaterial: Crystallization Behavior, Degradation, and in-Vitro Cellular Interactions. Adv. Funct. Mater..

[28] Han S.I., Yoo Y., Kim D.K., Im S.S. (2004). Biodegradable Aliphatic Polyester Ionomers. Macromol. Biosci..

[29] Li Y., Wang Z. (2025). Biomaterials for Corneal Regeneration. Adv. Sci..

[30] Ulag S., Ilhan E., Sahin A., Karademir Yilmaz B., Kalaskar D.M., Ekren N., Kilic O., Nuzhet Oktar F., Gunduz O. (2020). 3D printed artificial cornea for corneal stromal transplantation. Eur. Polym. J..

[31] Kim H., Kang N.-W., Hong W., Abdollahramezani S., Ngo G.-H., Raja D., Peters O., Mahajan V.B., Myung D., DeBoer C. (2026). Implantable ocular therapeutic systems: An insight into their clinical potential in the long-term treatment of ocular diseases. Biofabrication.

[32] Kanchan M., Tambe P.K., Bharati S., Powar O.S. (2024). Convolutional neural network for colorimetric glucose detection using a smartphone and novel multilayer polyvinyl film microfluidic device. Sci. Rep..

[33] Do T.C., Jeon H.J., Song H.H., Tashiro K., Kim Y.H., Ree M. (2010). Mesomorphic Phase in Oriented Poly(Pentamethylene 2,6-Naphthalate). Polymer.

[34] Jeong Y.G., Jo W.H., Lee S.C. (2002). Crystal Structure of Poly(Pentamethylene 2,6-Naphthalate). Polymer.

[35] (2022). Standard Practice for Computing the Colors of Objects by Using the CIE System.

[36] Glicerina V., Balestra F., Rosa M.D., Bergenhstål B., Tornberg E., Romani S. (2014). The Influence of Different Processing Stages on Particle Size, Microstructure, and Appearance of Dark Chocolate. J. Food Sci..

[37] Galus S., Lenart A. (2013). Development and characterization of composite edible films based on sodium alginate and pectin. J. Food Eng..

[38] Syahidad K., Rosnah S., Noranizan M.A., Zaulia O., Anvarjon A. (2015). Quality changes of fresh cut cantaloupe (*Cucumis melo* L. var Reticulatus cv. Glamour) in different types of polypropylene packaging. Int. J. Res..

[39] Siracusa V., Genovese L., Ingrao C., Munari A., Lotti N. (2018). Barrier Properties of Poly(Propylene Cyclohexanedicarboxylate) Random Eco-Friendly Copolyesters. Polymers.

[40] (2009). Biological Evaluation of Medical Devices—Part 5: Tests for In Vitro Cytotoxicity.

[41] Global Alliance of Eye Bank Associations (2018). The Barcelona Principles: An Agreement on the Use of Human Donated Tissue for Ocular Transplantation, Research, and Future Technologies©. Cornea.

[42] Kulkarni S., Raj D., Reddy K.M., Vyas C.O. (2014). Sulfonated Co-Polyesters and Method for Manufacturing. U.S. Patent.

[43] Li Z., Chu Y., Huang Q., Jin X., Qiu Z., Jin J. (2024). Crystallization Behavior of Copolyesters Containing Sulfonates. Polymers.

[44] Song J.M., Wang X.S., Li J.Y., Liu Y., Zheng L.C. (2025). Structure–Property Relationships of Ionic Poly(Ethylene Terephthalate) (PET): Effect of Ion Content and Species. Polym. Chem..

[45] Saumer A., Mecking S. (2023). Recyclable and Degradable Ionic-Substituted Long-Chain Polyesters. ACS Sustain. Chem. Eng..

[46] Li X., Zheng W.Z., Xu P.Y., Zhang Z.Y., Wang P.L., Lu B., Huang D., Zhen Z.C., Zhao Y., Ji J.H. (2024). Water-Soluble Biodegradable Polyesters with PH and Ionic Responsivity. J. Hazard. Mater..

[47] Zhang S.X., Wu X.L., Hao T.H., Hu G.H., Jiang T., Zhang Q.C., Zhao H. (2018). Structure Design, Fabrication and Property Investigation of Water-Based Polyesters with Notable Surface Hydrophilicity. New J. Chem..

[48] Liang Q., Wang K., Jiang Y., Li G., Yang F., Cao Y., Xiang M. (2025). Investigation on Non-Isothermal Crystallization Kinetics of Polyethylene Terephthalate-Polyethylene Naphthalate Blends. Polymers.

[49] Haernvall K., Zitzenbacher S., Yamamoto M., Schick M.B., Ribitsch D., Guebitz G.M. (2017). Polyol Structure and Ionic Moieties Influence the Hydrolytic Stability and Enzymatic Hydrolysis of Bio-Based 2,5-Furandicarboxylic Acid (FDCA) Copolyesters. Polymers.

[50] Motta A., Seguini G., Perego M., Consonni R., Boccia A.C., Ambrosio G., Baratto C., Cerruti P., Lavorgna M., Tagliabue S. (2022). Sequential Infiltration Synthesis of Al_2_O_3_ in Biodegradable Polybutylene Succinate: Characterization of the Infiltration Mechanism. ACS Appl. Polym. Mater..

[51] Mori H., Hara M. (2018). Transparent biocompatible wool keratin film prepared by mechanical compression of porous keratin hydrogel. Mater. Sci. Eng. C.

[52] Tighsazzadeh M., Boateng J. (2024). Matrix hyaluronic acid and bilayer poly-hydroxyethyl methacrylate-hyaluronic acid films as potential ocular drug delivery platforms. Int. J. Biol. Macromol..

[53] Mahmood N., Zha D., Gullion S., Eletmany M.R., Jahan U.M., El-Shafei A., Gilger B.C., Gluck J.M. (2026). Surface modified electrospun scaffold supports iPSC-derived limbal stem cell function. npj Biomed. Innov..

