# Supplementary material for: Naphthalene-Based Aromatic Copolyesters Containing Polar Units with Improved Wettability and High Transparency for Biomedical Applications
Source: Polymers (Basel). 2026 Sep 11;18(18):2212. doi: 10.3390/polym18182212 (PMC13611180; doi:10.3390/polym18182212)
Supplement: Supplementary file 1 [file polymers-18-02212-s001.zip › polymers-4493051-supplementary.pdf]

SUPPLEMENTARY MATERIALS

Table S1. Molecular (^{1}H-NMR, I.V.), thermal (TGA, I scan DSC) and mechanical (stress-strain measurements) characterization data of PPeN and P(PeN_xPeSI_y) copolymers compared to those of other aromatic systems reported in the literature containing DMSI as co-unit. [a] From Ref. [22]; [b] From Ref. [23]; [c] From Ref. [24]

	^{1}H-NMR	I.V.	TGA		I SCAN DSC		STRESS-STRAIN		
	**SI real**	**η**	**T_{id}**	**T_{max}**	**T_g**	**T_m**	**E**	**σ_B**	**ε_B**
	**mol%**	**dl/g**	**°C**	**°C**	**°C**	**°C**	**MPa**	**MPa**	**%**
**P(PeN)**	0	0.53	386	407	46	-	807 ± 24	22 ± 2	44 ± 8
**P($PeN_{90}PeSI_{10}$)**	8	0.41	354	386	50	-	878 ± 57	39 ± 4	82 ± 30
**P($PeN_{80}PeSI_{20}$)**	15	0.25	330	362	52	-	1315 ± 84	41 ± 1	17 ± 5
**PET** [a]	0	-	-	436	78	253	-	-	-
**$PET_{90}SI_{10}$** [a]	8.3	-	-	439	58	167	-	-	-
**$PET_{80}SI_{20}$** [a]	20.2	-	-	437	56	-	-	-	-
**PEF** [b]	0	1.03	361	396	88.9	227.7	3200 ± 100	87 ± 3	3.7 ± 0.5
**$sPEF_9$** [b]	9.1	0.55	352	383	95.4	205.3	-	-	-
**PHT** [c]	0	0.75	371	403	11	148	539	26	16
**$PHT_{90}SI_{10}$** [c]	9.9	0.56	350	369	14	130	700	30	200
**$PHT_{80}SI_{20}$** [c]	19.4	0.57	346	364	19	-	759	31	170

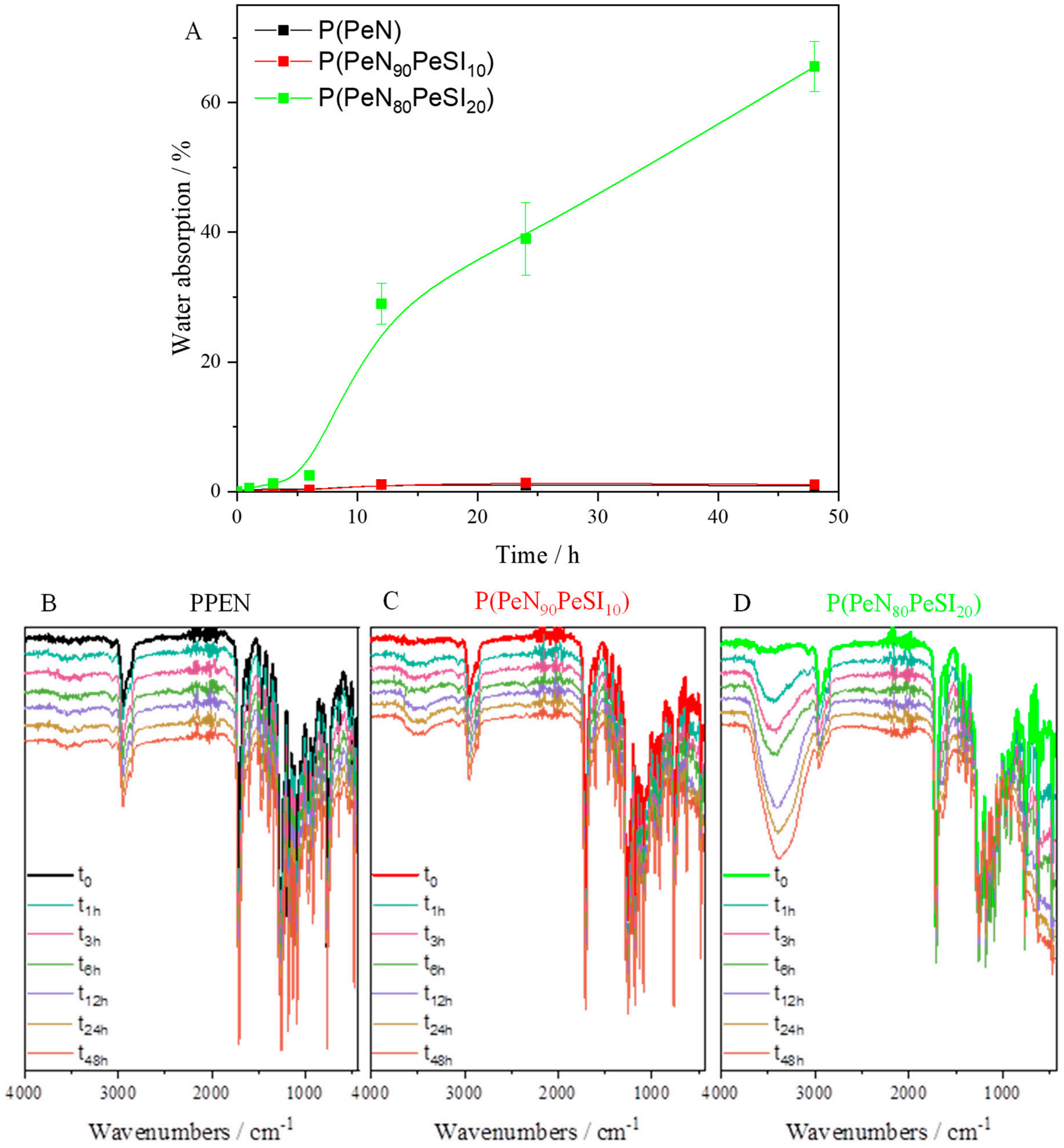

Figure S1. A) Quantitative water absorption data of PPeN and P(PeN_xPeSI_y) copolymers at different timepoints (1-48 h); FT-IR spectra before and after water immersion at different timepoints (1-48h) of B) of PPeN; C) P($PeN_{90}PeSI_{10}$) and D) P($PeN_{80}PeSI_{20}$).

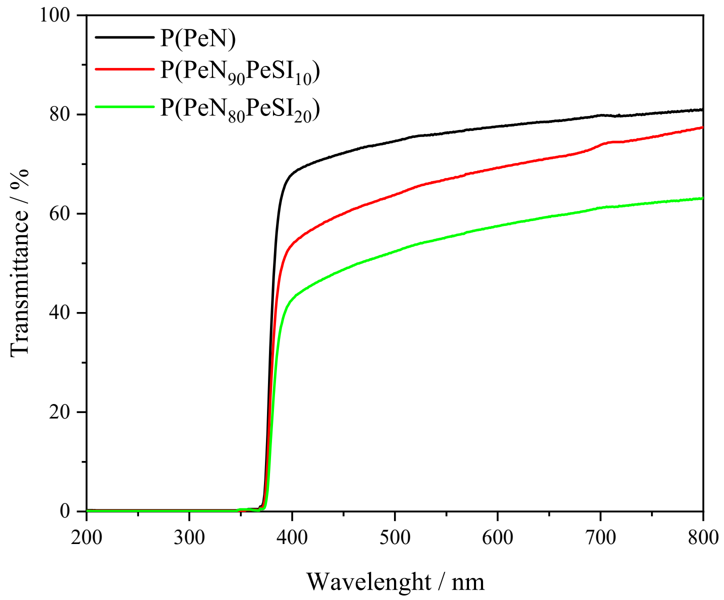

Figure S2. UV–vis light transmission spectra of PPeN and P(PeN_xPeSI_y) copolymer films (thickness of ~ 50 μm).
